# Supplementary material for: Hollow Mesoporous CeO2-Based Nanoenzymes Fabrication for Effective Synergistic Eradication of Malignant Breast Cancer via Photothermal–Chemodynamic Therapy
Source: Pharmaceutics. 2022 Aug 17;14(8):1717. doi: 10.3390/pharmaceutics14081717 (PMC9415169; doi:10.3390/pharmaceutics14081717)
Supplement: Supplementary file 1 [file pharmaceutics-14-01717-s001.zip › pharmaceutics-1829941-supplementary.pdf]

Hollow Mesoporous CeO_2-Based Nanoenzymes Fabrication for Effective Synergistic Eradication of Malignant Breast Cancer via Photothermal–Chemodynamic Therapy

Huaxin Tan [1], Yongzhen Li [1], Jiaying Ma [1], Peiyuan Wang [2,3], Qiaoling Chen [2,*] and Lidan Hu [1,*]

[1] Department of Biochemistry and Molecular Biology, The Key Laboratory of Ecological Environment and Critical Human Diseases Prevention of Hunan Province Department of Education, School of Basic Medicine, Hengyang Medical School, University of South China, Hengyang 421001, China

[2] Department of Anesthesiology, The First Affiliated Hospital of Xiamen University, Xiamen 361001, China

[3] Key Laboratory of Design and Assembly of Functional Nanostructures, Fujian Institute of Research on the Structure of Matter, Chinese Academy of Sciences, Fuzhou 350002, China

* Correspondence: mz1262585085@outlook.com (Q.C.); hulidan@usc.edu.cn (L.H.)

Part A: Supplementary Experimental Section

1. Experimental Section

1.1. Materials

Hexadecyltrimethylammonium bromide (CTAB, 99.99%), tetraethyl orthosilicate (TEOS, 98%), and (3-aminopropyl) triethoxysilane (APTES, 98%) were purchased from Sigma-Aldrich. NaOH (97%), cyclohexane (99.7%), and ethanol (99.5%) were obtained from Shanghai Chemical Co., Ltd. Ammonia aqueous solution (28 wt%), triethanolamine (TEA, 99.5%), methenamine (99%), and $Ce(NO_3)_3 \cdot 6H_2O$ (99.9%) were purchased from Aladdin Industrial Inc. All chemicals were used as received without further purification. In addition, 1,2-distearoyl-sn-glycero-3-phosphoethanolamine-N-amino (polyethylene glycol) (DSPE-PEG_{2000}-NH_2) was purchased from Shanghai Ponsure Biotech and RGD was supplied by GL Biochem (Shanghai) Ltd.

1.2. Instruments

Transmission electron microscopy (TEM) measurements were carried out on an H-7650 microscope (Japan) operated at 100 kV. Scanning electron microscope (SEM) measurement was analyzed using Nanoscope V multimode atomic force microscope. Photothermal images were obtained by FOTRIC 225s. The luminescence spectrums of all the samples were measured on an Edinburgh FLS-980 fluorescence spectrometer. Dynamic light scattering (DLS) and zeta potential were obtained on a NanoBrook Omni. UV-vis-NIR absorption spectra were measured on a Shimadz spectrophotometer (UV-3150) (Japan) with wavelength range of 300-1200 nm; unless

otherwise specified, all spectra were collected under identical experimental conditions. Confocal laser scanning microscope images were performed in Nikon A1RMP imaging system. In vivo NIR II fluorescent images were obtained under a NIR-OPTICS Series III 900/1700 system (808 nm laser irradiation with a 1000 nm long filter pass).

1.3. Statistical analysis

The data are presented as mean ± standard deviation. The significance of each group was analyzed by one-way analysis of variance (ANOVA). $*p < 0.05$ and $**p < 0.01$ indicate a significant difference and a highly significant difference between the parallel groups, respectively.

2. Methods

2.1 The pH-sensitive studies

First, pH = 5.6 and pH = 7.4 buffers were prepared. Then, 200 μg/mL $HCeO_2$ was added to the two types of buffer solutions for 0, 1, 2, and 4 h, respectively. The above samples were washed by 1X PBS (5000 rpm for 10 min). Finally, the ICG released in the supernatant was further detected by a fluorescence spectrometer. Meanwhile, the Ce ions and the morphology of $HCeO_2$ in precipitation were further analyzed by ICP-MS and TEM, respectively.

2.2. Photothermal effect investigations in vitro

Initially, 200 μg/mL of $HCeO_2$@ICG were placed into 200 μL tubes and illuminated by NIR laser (808 nm, 0.75 W cm^{-2}) for 0, 1, 2, 3, 4, and 5 min. Then, the

increased temperature and photothermal images of each time point were obtained by an infrared region camera (FOTRIC 225s). Meanwhile, $HCeO_2$ was set as the control. The exact temperature increase and photothermal images were acquired with the same procedures as $HCeO_2$@ICG.

2.3. Cell killing effect of synergistic therapy

Initially, 4T1 cells were seeded into a 6-well plate and $HCeO_2$@ICG-RGD was added for 24 h. Then, the cells were irradiated under 808 nm laser exposure for 5 min (0.75 W/cm^2). Finally, after another 12 h of incubation, cell viability was studied by the CCK-8 kit.

2.4. Live/dead cell discrimination and cell apoptosis evaluation

Both calcein-AM/PI and Annexin V-FITC/PI apoptosis assays were also used to evaluate the in vitro antitumor efficiency. Initially, 4T1 cells were seeded into a 1×10^5/well with 1 mL of 1640 fresh medium (10% FBS, 100 units/mL of penicillin, and 100 μg/mL of streptomycin) into a 6-well plate for 24 h. Then, the cells were treated by different formulations (PBS, ICG+laser, $HCeO_2$@ICG-RGD, $HCeO_2$@ICG-RGD+laser). Laser treated groups were irradiated under 808 nm laser exposure for 5 min (0.75 W cm^{-2}). All treatment groups were stained by calcein-AM/PI assay and the resultant four samples were imaged by CLSM. For the Annexin V-FITC/PI apoptosis assay, PBS, ICG+laser, $HCeO_2$@ICG-RGD, $HCeO_2$@ICG-RGD+laser treated groups were stained by Annexin V-FITC and PI, and the percentage of apoptosis/necrosis was detected by a flow cytometer.

Part B: Supplementary Figures

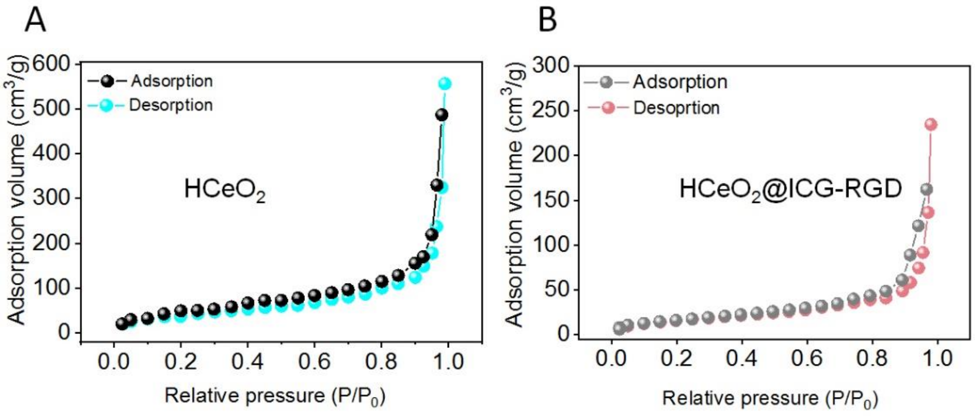

Figure S1. Nitrogen adsorption–desorption isotherms of $HCeO_2$ (A) and $HCeO_2$@ICG-RGD (B).

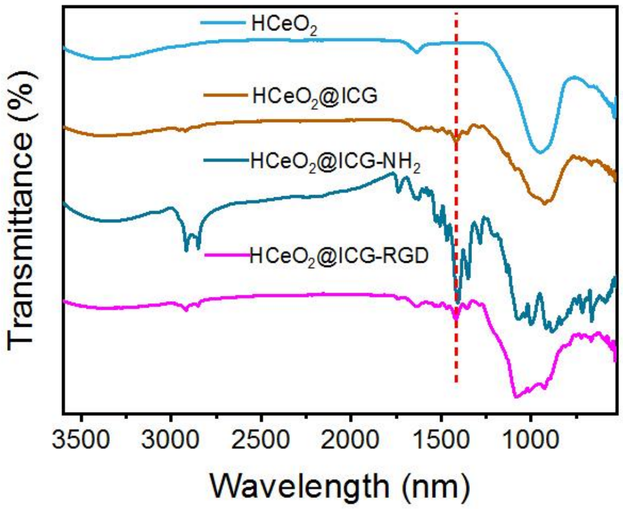

Figure S2. FTIR spectra of $HCeO_2$, $HCeO_2$@ICG, $HCeO_2$@ICG-NH_2, and $HCeO_2$@ICG-RGD.

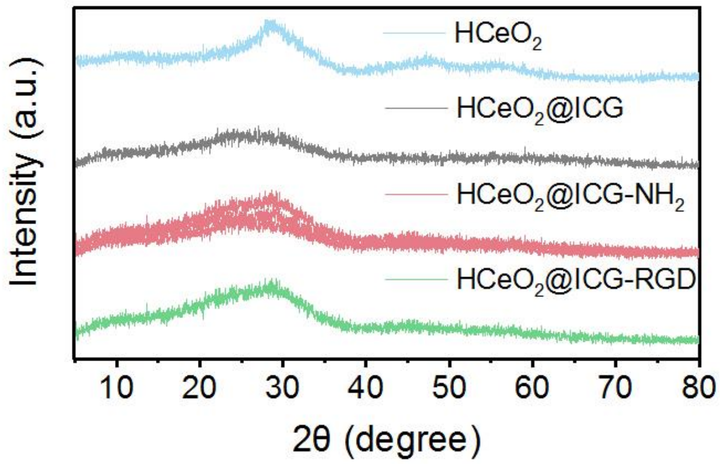

Figure S3. XRD spectra of $HCeO_2$, $HCeO_2$@ICG, $HCeO_2$@ICG-NH_2, and $HCeO_2$@ICG-RGD.

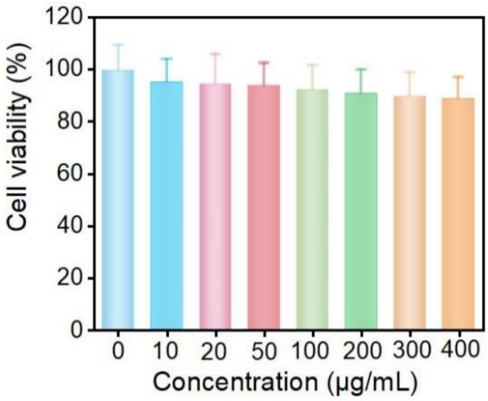

Figure S4. Cell viability of normal cells (HUVEC) after 24-h treatment of $HCeO_2$@ICG-RGD.

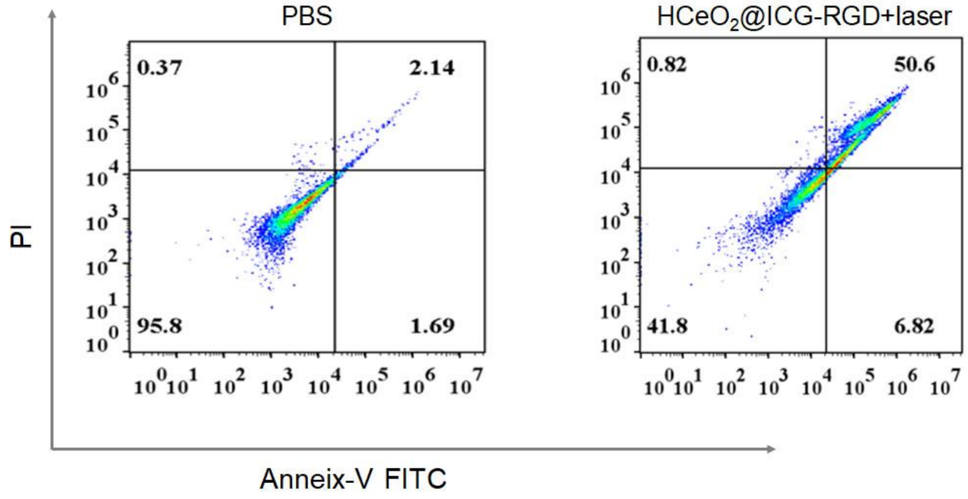

Figure S5. Cell apoptosis analyzation of PBS and HCeO$_2$@ICG-RGD+laser treated 4T1 cells via Annexin-V FITC and PI staining.

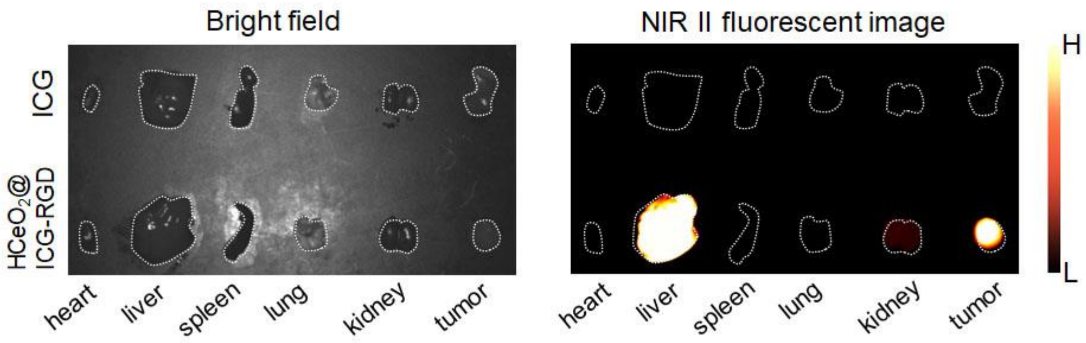

Figure S6. Ex vivo bright field image (left) and NIR II fluorescent image of major organs (heart, liver, spleen, lung, kidney) and tumors after tail vein injection of HCeO$_2$@ICG-RGD for 24 h.

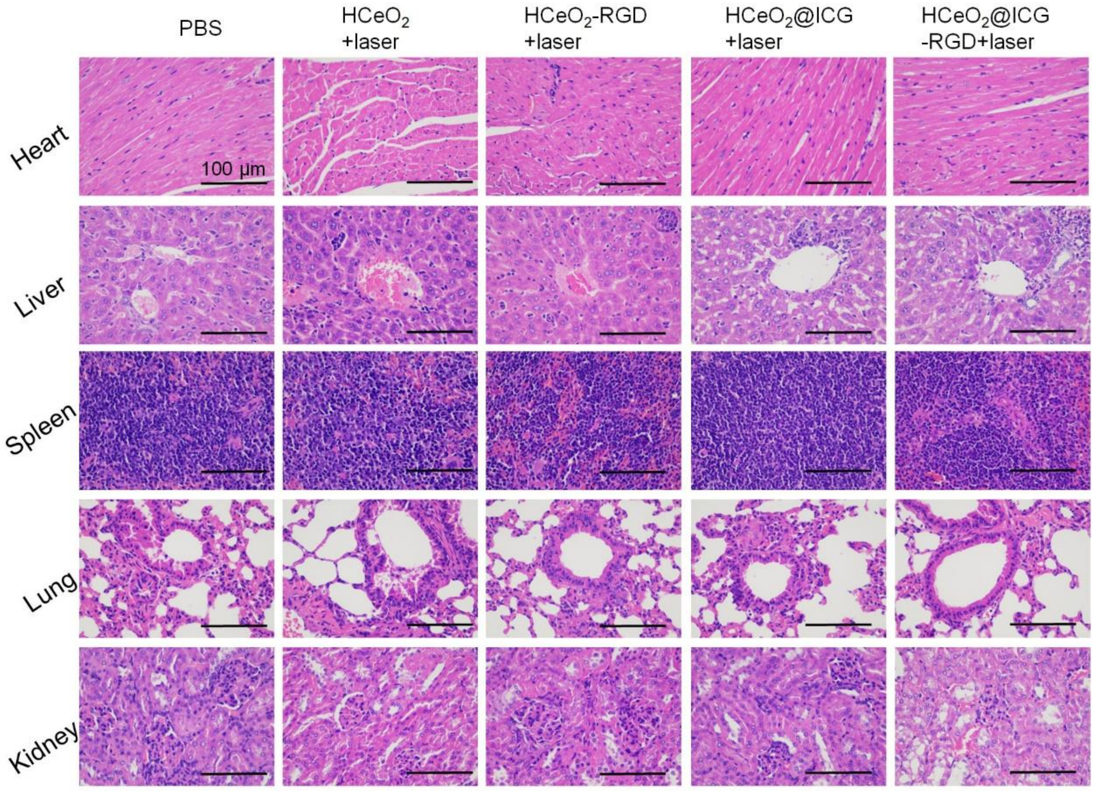

Figure S7. H&E staining images of major organs (heart, liver, spleen, lung, kidney) from breast tumor-bearing mice after different treatments (PBS, $HCeO_2$+laser, $HCeO_2$@ICG+laser, $HCeO_2$@ICG+laser, and $HCeO_2$@ICG-RGD+laser) for 18 days.
